# Adipocytes in Aortic Stenosis: Association With Clinical and Morphological Indices

**DOI:** 10.1002/ca.70045

**Published:** 2025-11-09

**Authors:** Elena Zoico, Tanaz Saatchi, Silvia Urbani, Vanni Rizzatti, Gloria Mazzali, Francesco Fantin, Silvia Faccioli, Alessandro Gavras, Mauro Zamboni, Anna Giani

**Affiliations:** ^1^ Division of Geriatric Medicine, Department of Medicine University of Verona Verona Italy; ^2^ DIPSCO, Department of Psychology and Cognitive Science CISMed University of Trento Trento Italy

**Keywords:** adipocytes, aging, aortic stenosis

## Abstract

Recently, great attention has been given to understanding the new pathogenetic mechanisms underlying aortic stenosis (AS). The study aims to understand the role of mature adipocytes in AS and their association with histologic, clinical, and echocardiographic data, an area previously overlooked in AS research. We enrolled 25 patients (15 women and 10 men) with severe AS undergoing elective aortic valve replacement. Each patient underwent clinical and transthoracic echocardiographic evaluations before surgery. We obtained AS valves and left ventricular (LV) septal biopsies to assess the presence of adipocytes within the valve using perilipin 1 (PLIN1) immunohistochemistry, and we also examined other histological characteristics of the ventricular biopsies. Adipocytes were detected in 76% of the aortic stenotic valve samples, often grouped adjacent to calcified areas. Patients with higher values of PLIN1 valvular adipocytes were generally older (*p* = 0.06) and had lower BMI values (*p* = 0.06). Moreover, the group with a higher presence of PLIN1(+) valvular adipocytes had significantly decreased mean gradient values and reduced M1 macrophage infiltration in ventricular biopsies. In a binary regression analysis, only mean gradient was significantly associated with the presence of PLIN1(+) adipocytes in the valve, regardless of age, BMI and ventricular M1 macrophage levels. These preliminary findings suggest that valvular adipocytes could be related to the progression of AS, but more investigation is necessary.

## 1 Introduction

Aortic stenosis (AS) is the leading valvular heart disease in the aging population (Iung and Vahanian 2011). This condition is marked by progressive thickening and calcification of the aortic valve cusps, leading to sclerosis and advanced valve dysfunction with associated inflammatory, atherogenic, and osteogenic changes (Freeman and Otto 2005; Rajamannan et al. 2007). AS also impacts the left ventricle, affecting ventricular compliance and leading to myocardial fibrosis, ventricular dilation, and heart failure (Spilias et al. 2022; Chatterjee et al. 2023). Currently, no pharmacological treatments have proven effective in slowing AS progression, making surgical valve repair the primary treatment option.

It had been hypothesized that the pathophysiology of AS shared common mechanisms with atherosclerosis as both conditions share common risk factors such as obesity, diabetes, metabolic syndrome, and hyperlipidemia (Abdul‐Rahman et al. 2023). According to this hypothesis, the damage begins with acute endothelial injury, deposition of oxidized lipid and consequent inflammation and activation of valvular interstitial cells (VICs), ultimately leading to calcification and fibrosis of the valve (Olszowska 2011).

Recently, Farrar et al. (Farrar et al. 2021) demonstrated the importance of developmental processes in the pathogenesis of AS, such as the endothelial to mesenchymal transition (EMT) that leads valve endothelial cells (VECs) to assume a chondro‐osteogenic phenotype associated with aortic valve calcifications.

The presence of adipocytes in the aortic valve has been a subject of limited but significant study. Fat tissue is typically found in the heart's epicardium, pericardium, and interatrial septum; however, it is not normally present in the heart valves and its infiltration in cardiac valves is exceptionally rare (Matloch et al. 2016; Al‐Makhamreh et al. 2023; Otto et al. 1994).

Matsukuma et al. (2013) found that the presence of adipocytes in the spongiosa layer between the fibrosa and ventricularis correlates with age, implying an age‐dependent accumulation that might increase the risk of AS in older populations. A study by A. Cipriain et al. (2020), indicates a positive correlation between the presence of adipocytes in the aortic valve and valve thickness, as measured by echocardiogram, suggesting a potential link between adipocyte infiltration and changes in valve structure.

Although there are several studies of adipocytes within cardiac valves, the comprehensive characterization of these cells in the valves, the mechanisms and the significance of this phenomenon remain largely speculative.

This preliminary study aims to characterize adipocytes in AS valves and explore their association with histologic, clinical, and echocardiographic data.

## 2 Methods

### 2.1 Study Population

Briefly, from a wider group of subjects undergoing elective surgical valve replacement for severe AS, we considered for these analyses 25 patients (10 males, 15 females) who underwent comprehensive assessments and analysis of the aortic valve. Subjects with significant coronary artery disease were excluded, as well as patients with recent unintentional weight loss, treatment with steroids, immunosuppressants, or hormones, and certain chronic health conditions (for more details, please refer to the previously published work, Zoico et al. 2023). All participants provided informed consent and the study was approved by the University of Verona's Ethical Committee (protocol number: 381CESC; approval date: December 23, 2014). Participant demographics and main diseases and medications are detailed in Table 1.

**TABLE 1:** Comparison of the main clinical, anthropometrical, and biomolecular characteristics between female and male patients with severe aortic stenosis enrolled in the study.

	Males (mean ± SD) *n* = 10	Females (mean ± SD) *n* = 15	Significance
Age (years)	73.9 ± 4.9	72.0 ± 6.5	NS
Weight (kg)	84.2 ± 10.3	74.5 ± 13.8	NS
Height (cm)	173.0 ± 4.9	163.1 ± 4.4	NS
BMI (kg/m^2^)	28.2 ± 2.9	27.9 ± 4.7	NS
Waist circumference (cm)	101.3 ± 8.6	97.7 ± 13.8	NS
Systolic blood pressure (SBP) (mmHg)	136.0 ± 16.3	135.1 ± 18.7	NS
Diastolic blood pressure (DBP) (mmHg)	68.0 ± 9.2	70.4 ± 8.6	NS
Heart rate (HR) (bpm)	70.4 ± 13.3	71.7 ± 8.5	NS
Blood glucose (mg/dl)	108.7 ± 34.7	119.0 ± 37.0	NS
Insulin levels (mU/L)	10.9 ± 9.1	6.5 ± 3.7	NS
HOMA index	3.5 ± 3.9	2.1 ± 1.7	NS
Total cholesterol (mg/dl)	170.0 ± 38.4	178.9 ± 43.4	NS
HDL (mg/dl)	48.6 ± 8.5	64.5 ± 18.1	NS
LDL (mg/dl)	87.1 ± 33.2	96.8 ± 31.6	NS
Triglycerides (TG) (mg/dl)	134.8 ± 75.6	94.6 ± 33.6	NS
Albumin (g/dl)	38.8 ± 2.9	38.5 ± 2.7	NS
Creatinine (μmol/L)	95.9 ± 30.8	70.0 ± 12.9	NS
Hemoglobin (g/dl)	13.5 ± 1.9	13.5 ± 1.2	NS

Abbreviations: BMI, body mass index; HDL, high density lipoprotein cholesterol; HOMA index, homeostatic model assessment; LDL, low density lipoprotein cholesterol; NS, nonsignificant; SD, standard deviation.

### 2.2 Bio‐Clinical and Anthropometric Evaluation

Subjects underwent clinical evaluation, including blood pressure and heart rate measurement. Data on smoking, existing diseases and medication use were collected. They gave venous blood samples after fasting for metabolic tests. Plasma glucose levels were measured using a Beckman Instruments glucose analyzer. Cholesterol and triglyceride levels were assessed with a Techincon Auto Analyzer, and HDL was derived using dextran‐magnesium precipitation. Subjects' weight (Salus Scale) and height (Salus Stadiometer) were recorded, and BMI was calculated as weight in kg divided by height in m^2^. Waist circumference was also measured at the abdomen's narrowest point as previously detailed (Zoico et al. 2023). Additionally, we measured the plasma levels of adiponectin, one of the main adipokines secreted by adipocytes.

### 2.3 Echocardiographic Assessment

The entire study population underwent preoperative echocardiography using a Philips IEE echocardiograph with speckle tracking analysis, performed in the echocardiography clinic of the Cardiology Department of the Integrated University Hospital of Verona. For each individual, we assessed left ventricular systolic function (ejection fraction, EF, using the biplane Simpson method %), mean transvalvular aortic gradient, and peak transvalvular gradient. Additionally, diastolic function indices were analyzed using tissue Doppler, including the E/A ratio (ratio of E wave, early diastolic filling, to A wave, atrial diastolic filling). Ultimately, LV Global Longitudinal Strain (GLS) was measured as an index of systolic function using a two‐dimensional method.

### 2.4 Sample Collection

In the operating room, following a median sternotomy and approximately 1 h postanesthesia induction, myocardial samples (from the interventricular septum) and stenotic aortic valves were obtained; all examined valves in our study were trileaflet. The biopsies were then preserved for subsequent immunohistochemical (IHC) analysis.

### 2.5 IHC Analysis of Adipocytes in Aortic Valves and Ventricles

Freshly isolated aortic valve and ventricular myocardial samples were fixed in 4% paraformaldehyde. Following fixation, aortic valve tissues were placed in Osteodec solution (Bio‐Optica, Italy) for 16 h. This solution, primarily containing EDTA bisodium, acts as a chelating agent to remove calcium deposits while maintaining the structural integrity of the tissues. After decalcification, the tissues underwent three washes in PBS ensuring thorough removal of the decalcifying agent. Subsequently, the tissues were hydrated and embedded in paraffin. In our study, the histological analysis was focused on the cusps of the aortic valves, the main zone of stenosis‐related changes like thickening and calcification. Five micrometer sections were stained with hematoxylin and eosin for morphological evaluation before immunohistochemistry. Human Perilipin‐1 antibody was the primary antibody for detecting adipocytes in valve and ventricle samples. Signal Stain Boost IHC HRP Rabbit reagent was the secondary antibody. A negative control using only the secondary antibody was also conducted. For IHC, the DAB peroxidase substrate kit was used. Sections underwent deparaffinization, antigen retrieval in sodium citrate buffer and blocking of endogenous peroxidase and nonspecific binding. After incubation with primary and secondary antibodies, DAB was applied for peroxidase activity visualization. Sections were counterstained with hematoxylin, mounted with Entellan and covered with coverslips. A comprehensive protocol has been previously detailed (Zoico et al. 2023).

### 2.6 Image Acquisition and Analysis

Advanced imaging systems, including the Olympus BX51 photomicroscope and EVOS FL Auto Cell Imaging System were used to obtain high‐magnification examination of valve and ventricular sections. Manual quantification of PLIN1‐positive adipocytes was examined across the full tissue area, with adipocyte density expressed in cells/mm^2^. This process confirmed comprehensive analysis and accurate representation of adipocyte infiltration in the samples. For an in‐depth methodology, please refer to our earlier publication (Zoico et al. 2023).

### 2.7 Additional Histological or IHC Analyses on Ventricular Biopsies

Ventricular myocardial muscle samples were fixed in 4% paraformaldehyde, dehydrated, cleared, and paraffin embedded. Sections of five micrometers were prepared for IHC analysis. The sections underwent antigen retrieval, endogenous peroxidase and biotin/avidin activity blocking and nonspecific binding prevention. Primary antibodies for macrophage infiltration analysis (CD68, IDO, and CD163, respectively for total, M1, M2 macrophages) were used. After overnight incubation with primary antibodies, sections were treated with biotinylated secondary antibodies and Vectastain ABC reagent (VectorLab). Negative controls used only secondary antibodies. Subsequently, sections were stained with diaminobenzidine (DAB) for peroxidase activity visualization and counterstained with hematoxylin, followed by mounting with coverslips. For a detailed explanation of our methodology, please consult our previous publication (Zoico et al. 2023, 2017).

### 2.8 Statistical Analysis

Descriptive data are presented as mean ± SD. Non‐normal variables were log‐transformed before analyses. Independent samples *t* tests were used to compare the main characteristics between female and male patients. Fisher's exact test (*χ* ^2^) was used to compare the main disease between women and men enrolled in the study. Independent samples *t* tests were also used to compare the main characteristics between patients with valvular PLIN1 values above and below the median value.

Binary regression analysis was performed to evaluate the effects of independent variables on valvular PLIN1 adipocytes. A *p* < 0.05 was used for statistical significance. All analyses were performed using SPSS (software version 17.0, SPSS).

We did not use Artificial Intelligence to develop any part of the manuscript.

## 3 Results

As detailed in Table 1, the study sample consisted of 15 females and 10 male patients. Given the absence of differences in age, anthropometric and bio‐clinical characteristics between genders in this preliminary sample, both female and male subjects were collectively analyzed (Table 1).

**FIGURE 1:**
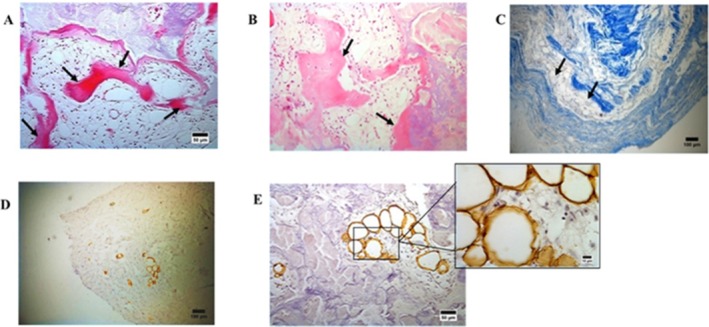
Morphological overview of aortic stenotic valves. (A and B) H/E staining reveals lamellar bone structures, identificated with arrows (100× magnification, scale bar = 50 μm). (C) Masson's Trichrome staining displays collagen derangement and fragmentation in blue (100× magnification, scale bar = 50 μm). Lipid infiltration evaluated via IHC using the primary anti‐perilipin 1 antibody (PLIN1, recognized as an adipocyte‐specific perilipin). (D) Identification of PLIN1 adipocytes within the valve leaflet (100× magnification, scale bar = 100 μm). (E) PLIN1+ adipocytes adjacent to areas of calcification (100× magnification, scale bar = 50 μm) and an in‐depth view into their detailed morphology (400× magnification, scale bar = 10 μm).

Morphological analysis of aortic stenotic valves showed lamellar bone structures on the leaflets, as evidenced by H/E staining (Figure 1A,B). Additionally, Masson's Trichrome staining highlighted collagen derangement and fragmentation in blue (Figure 1C). Adipocyte infiltration in AS valves was evaluated via IHC using the primary anti‐perilipin 1 antibody (recognized as an adipocyte‐specific perilipin) (Figure 1D,E).

**FIGURE 2:**
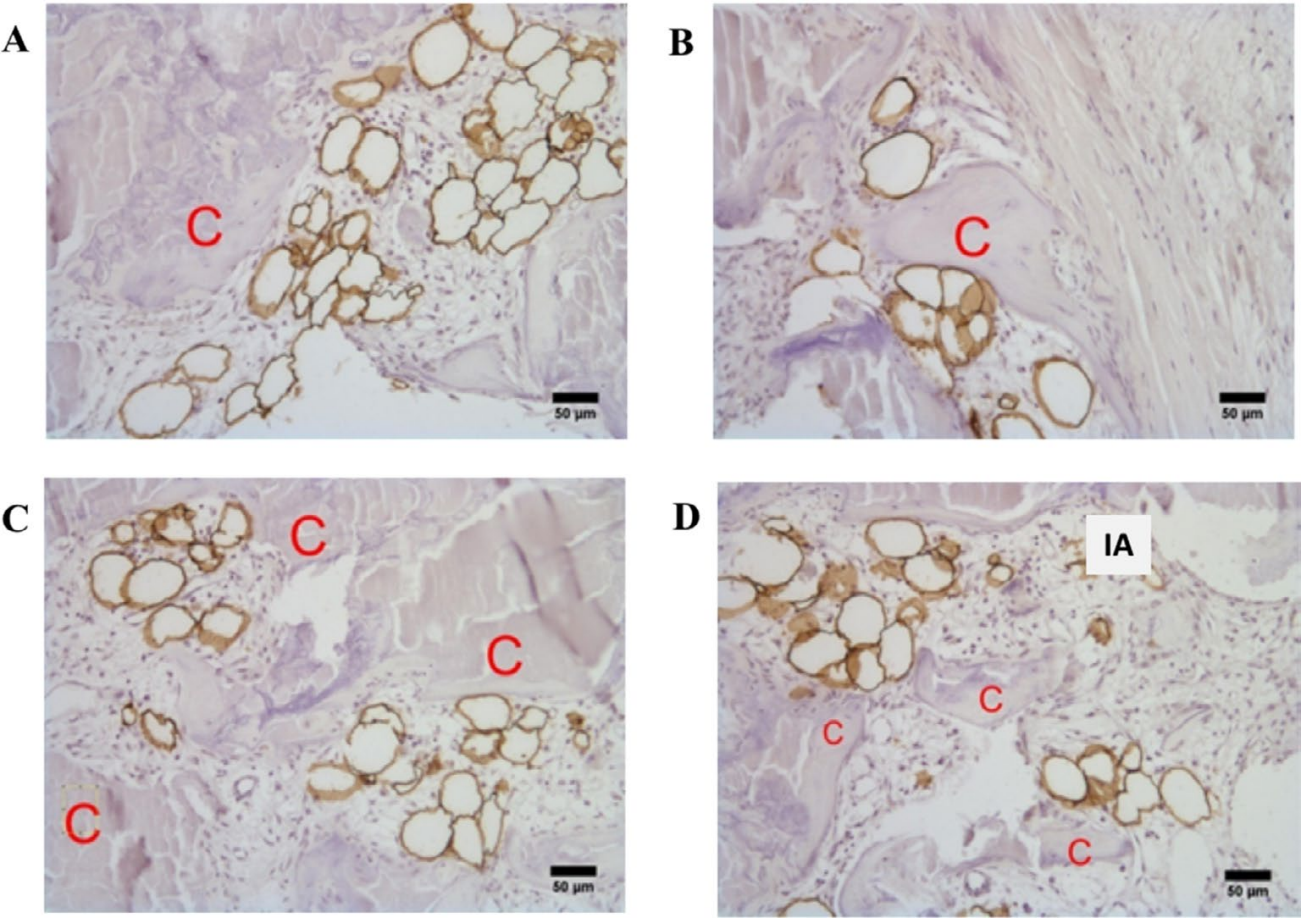
Localization of adipocytes in aortic stenotic valves. Localization of adipocytes (perilipin 1 positive cells [PLIN1+ cells]) near calcified regions (C). Following IHC with the PLIN1 antibody and counterstaining with Mayer's Hematoxylin, the brown‐stained adipocytes are observed adjacent to calcified regions (A–D). Isolated adipocytes (IA) can also be observed in (D) (100× magnification, scale bar: 50 μm).

Adipocytes' infiltration was detected in 76% of the valves (19 out of 25), with an average density of 3.32 adipocytes/mm^2^, ranging from 0.15 to 14.94/mm^2^. PLIN1(+) adipocytes were found to aggregate in small groups and were rarely isolated in the extracellular matrix (Figure 1D,E). In particular, following IHC using the PLIN1 antibody and subsequent counterstaining with Mayer's Hematoxylin, the brown‐stained adipocytes appeared in groups, closely connected to inflammatory cellular elements and, in particular, adjacent to calcified areas (Figure 2). Moreover, adipocytes were in tight association with cells with the characteristics of smooth muscle cells or myofibroblasts (Figure 2).

**FIGURE 3:**
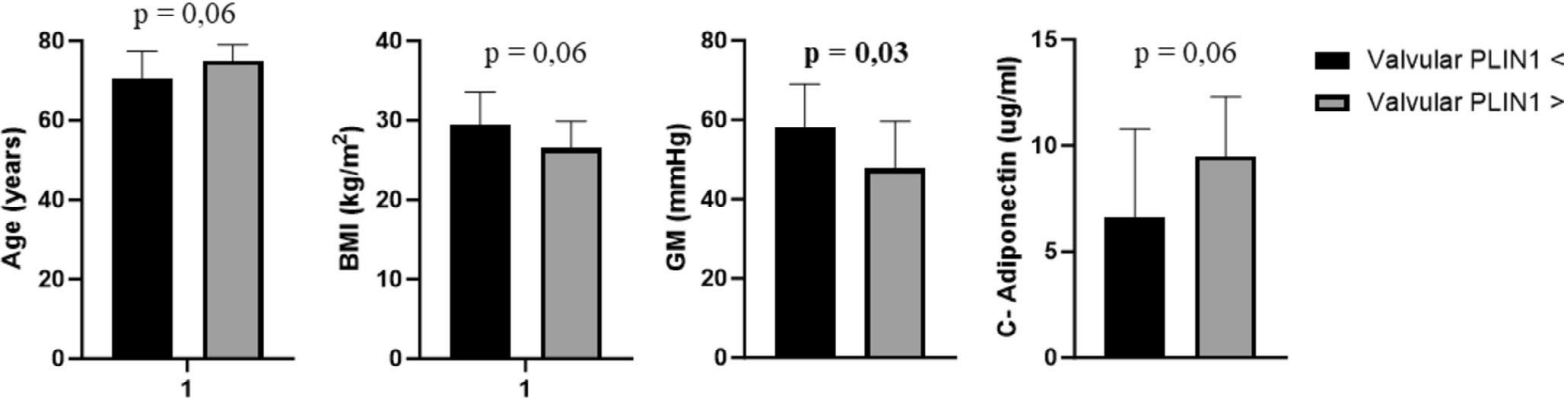
Comparison of mean values for key study variables based on valvular PLIN1 adipocyte categories. The categories were determined by whether valvular PLIN1 adipocytes were lower or higher than the median value (Valvular PLIN1 < or ≥ median value). The figure displays only the statistically significant variables. BMI, body mass index; C‐adiponectin, circulating adiponectin; GM, mean valvular gradient.

We divided the study sample according to the presence of PLIN1(+) adipocytes. The group with higher values of PLIN1(+) adipocytes tended to be older (*p* = 0.06) and had lower BMI values (*p* = 0.06) (Figure 3). No other significant differences were found in clinical and biohumoral variables. Moreover, the group with higher PLIN1(+) adipocytes presented significantly decreased mean gradient values (Figure 3).

Regarding the histological characteristics of the ventricular biopsies, only the amount of M1 macrophages was weakly correlated with PLIN1(+) adipocytes in the valve (Figure S1). Specifically, patients with elevated adipocyte valvular infiltration demonstrated reduced M1 macrophage levels in ventricular biopsies. Other histological characteristics of the ventricular samples, such as the degree of fibrosis or lipid infiltration (evaluated by IHC for PLIN5 and ceramides), did not show any significant association with valvular PLIN1(+) adipocytes (data not shown). Finally, significantly higher circulating levels of adiponectin characterized subjects with a higher number of adipocytes in the valve (Figure 3). However, after adjusting for BMI values, the correlation between adiponectin and PLIN1(+) adipocytes was no longer significant (data not shown).

Subsequently, we performed a binary regression analysis to explore the relationship between the number of valvular PLIN1(+) adipocytes (categorized as high vs. low adipocyte count) and different independent variables, including age, BMI, mean valvular gradient, and ventricular M1 macrophages (Table S1). Within this model, the mean gradient was the only variable significantly associated with PLIN1(+) adipocytes in the valve (*p* = 0.036), independent of other confounding factors (Table S1).

## 4 Discussion

Adipocytes were not initially identified in aortic valves; until 1990–2000, only lipid deposits or accumulations had been described in AS. Matsukuma et al. (2013), identified various stages of adipocyte maturation as well as forms of adipocytic degeneration in different types of dysfunctional aortic valves. In that clinicopathological study, the authors raised the question of whether valvular adipocytes might be associated with aortic valvular dysfunctions (Matsukuma et al. 2013). However, since then, the question remains unanswered.

Utilizing immunohistochemistry with an antiperilipin antibody, which is specific to adipocytes, we observed a high presence of adipocytes in AS valves, with adipocyte infiltration noted in 76% of the valves. In the literature, the proportion of valves affected by adipocyte presence varies depending on the type of data sample, the characteristics of valvular dysfunctions (such as stenosis, regurgitation, or a combination of both), and the techniques used. It ranges from 50% to 70% (Matsukuma et al. 2013; Pichler Sekulic and Sekulic 2021) to only 16% in cases where adipocytes represent a metaplastic condition (Galli et al. 2017).

Fat cells and fat‐related lesions are associated with older age, according to several reports (Matsukuma et al. 2013; Pichler Sekulic and Sekulic 2021; Galli et al. 2017). It has been observed that the average age of patients with adipocytes in their aortic valves was greater compared to patients without valvular adipocytes. Additionally, we found that the group with higher levels of PLIN1(+) adipocytes tended to be older, confirming a possible age‐related increase in adipocytes within stenotic valves.

In contrast to other deposits of cardiac fat, we did not find any association between adipocytes and BMI, obesity, or other indices of fat distribution, nor did we find a link with any other concomitant disease, in line with previous studies (Matsukuma et al. 2013; Pichler Sekulic and Sekulic 2021; Galli et al. 2017). However, the limited number of patients enrolled in these studies precludes the ability to draw definitive conclusions. To explore the relationship between obesity and adipocyte infiltration in AS, further studies are mandatory.

Intriguingly, our analysis revealed an inverse relationship between the presence of adipocytes in aortic valves and transvalvular gradients, confirming the findings of Pichler Sekulic et al. (Pichler Sekulic and Sekulic 2021), who observed lower gradients in stenotic valves with more adipocytes.

Even though this is only a descriptive study, the relationship observed between age and valvular adipocytes, as well as between the transvalvular gradient and fat cells in aortic stenotic valves, could be significant enough to speculate on some clinicopathological aspects.

The association with age is relevant because as aging occurs, the known risk factors for atherosclerotic lesions increase. In recent years, AS and atherosclerosis have often been considered similar disorders (Abdul‐Rahman et al. 2023). According to this hypothesis, the process begins with endothelial injury and the deposition of oxidized lipids into the valves. These processes cause inflammation and activate vascular interstitial cells (VICs), ultimately leading to the calcification and fibrosis of the valve (Abdul‐Rahman et al. 2023). However, some authors have observed that signs of lipid accumulation, a significant component of atherosclerotic plaque, are rarely observed in the histopathological changes that precede AS (Galli et al. 2017). These observations put the basis for other mechanisms for valvular remodeling typical of AS.

The inverse relationship between valvular adipocytes and the transvalvular gradient suggests that adipocytes are more prevalent in less stenotic aortic valves, which experience less shear stress. Mechanical stress has been identified as a key initiating step in the process of AS, favoring inflammation and activation of different types of cells involved in valvular damage. In fact, mechanical stress, at the tissue level activates VICs inducing myofibroblast activation and fibrosis degeneration of the valve as well as stimulating osteoblastic differentiation and valvular calcification. However, in the last years, great relevance, has been revolved also to endothelial‐to‐mesenchymal transition (EMT) (Aikawa and Hutcheson 2022). It has been hypothesized that, as a response to a disrupted communication with VIC, VEC adopt a chondro‐osteogenic phenotype, associated with calcification and collagen deposition in the valves (Farrar et al. 2021; Aikawa and Hutcheson 2022). On a cellular level, biomechanical stress activates mechano‐transduction pathways such as TGF‐beta and nuclear factor‐kB (Anousakis‐Vlachochristou et al. 2023). Additionally, VIC‐VEC co‐cultures are sensitive to increased shear stress and VECs exposed to a stiff matrix show an increase in the EMT phenomenon (Anousakis‐Vlachochristou et al. 2023). Mesenchymal valvular stem cells have the capacity to differentiate into both osteoblasts and adipocytes (Galli et al. 2017). Thus, it is possible to hypothesize that under conditions of reduced mechanical stress, adipocytes are more prevalent in the valve than osteoblasts.

Finally, the origin of fat cells is not clear: they likely arise from perivascular pluripotent cells (Brooks and Perosio 2012). However, the absence of vascularization in the valve suggests that fat cells probably originate from mesenchymal stem cells that are normally present in the valve (Brooks and Perosio 2012). From this perspective it is interesting to note that under specific culture conditions, VICs from valvular cusps could differentiate in vitro into an adipogenic direction (Galli et al. 2017).

The role of valvular adipocytes is likewise to be fully characterized. A recent study demonstrated that pioglitazone, a PPARγ activator, was capable of reducing the calcific degeneration of the aortic valve in diabetic rats (Katahira et al. 2021). This effect was observed through the reduction of VIC transformation into osteoblasts, probably favoring their conversion into adipocytes. This is of course a speculative hypothesis that needs to be evaluated in future studies.

We must acknowledge some limitations in the present study. First, the preliminary nature of the observations reported needs further confirmation in a wider population sample. Second, the lack of a control group of patients with normal valves, that is always difficult to obtain, is a notable limitation. Third, the observational nature of the study limits it to show associations without exploring the underlying mechanisms. For this purpose, it could be interesting to study other histologic aspects in stenotic aortic valves and their relationship to adipocytes, such as the presence of other cell types (such as myofibroblasts, smooth muscle cells) or the degree of inflammation and the presence of neovascularization. Moreover, proteomic data may also be useful to study the mechanisms supported by the presence of adipocytes and possibly involved in the pathogenesis of aortic valvular degeneration. Altogether, these analyses should be helpful in better assessing the impact of the presence of adipocytes in aortic stenotic valves.

## 5 Conclusion

In conclusion, our preliminary study shows that, in a small sample of elderly subjects with AS, valvular adipocytes are more prevalent in patients with lower mechanical stress on the valve, independently of age, BMI and ventricular inflammatory macrophage levels. These preliminary findings suggest that valvular adipocytes could play a role in the progression of AS, but more investigation is necessary, especially to explore the relation of adipocytes with other cells in the pathogenetic mechanisms of AS. The full characterization of valvular adipocytes needs to be completed to explore their role in aortic valvular degeneration, with the final goal of identifying new targets for treating the disease.

## Ethics Statement

The study was conducted in accordance with the Declaration of Helsinki and approved by the Ethics Committee of the University of Verona (protocol number: 381CESC; approval date: December 23, 2014).

## Consent

Informed consent was obtained from all subjects involved in the study.

## Supporting information


**Figure S1:** ca70045‐sup‐0001‐FigureS1.tiff.

**Table S1:** ca70045‐sup‐0002‐TableS1.tif.

## Data Availability

The data that support the findings of this study are available from the corresponding author upon reasonable request.
