# Supplementary figures and images for: Adipocytes in Aortic Stenosis: Association With Clinical and Morphological Indices
Source: Clin Anat. 2025 Nov 9;39(3):325–31. doi: 10.1002/ca.70045 (PMC12988313; doi:10.1002/ca.70045)

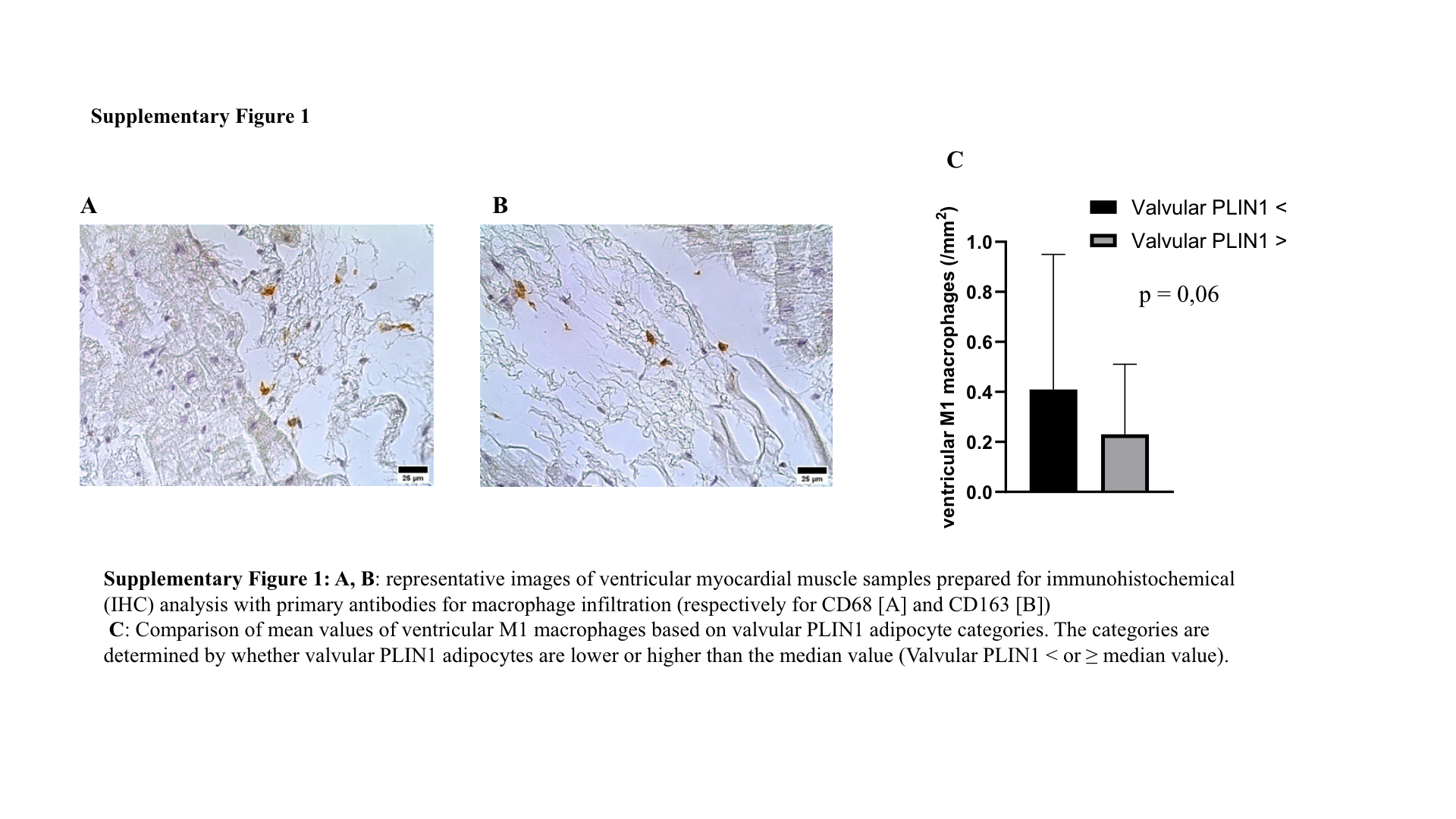

Supplement: Supplementary file 1 — Figure S1: ca70045‐sup‐0001‐FigureS1.tiff. [file CA-39-325-s001.tiff]

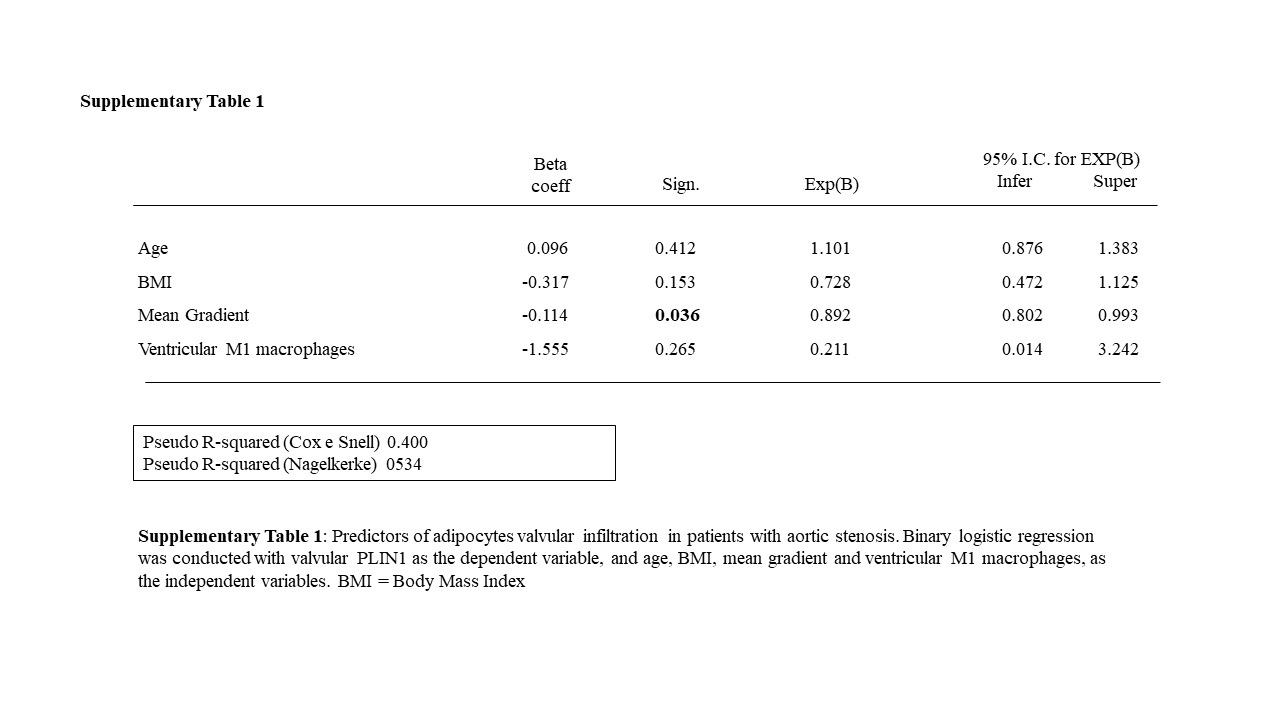

Supplement: Supplementary file 2 — Table S1: ca70045‐sup‐0002‐TableS1.tif. [file CA-39-325-s002.tif]
